# Targeting the role played by microbiota and adaptive immunity within autoimmune disease complexity

**DOI:** 10.3389/fimmu.2025.1718417

**Published:** 2025-12-01

**Authors:** Anders Bredberg, Katrine R. Nermo, Gunnel Henriksson

**Affiliations:** 1 Department of Medical Microbiology, Innlandet Hospital Trust, Lillehammer, Norway; 2 Department of Laboratory Medicine, Lund University, Lund, Sweden

**Keywords:** microbiota, adaptive immunity, autoimmune disease, intestinal microbiota, autoimmune disease complexity

## Introduction

All human individuals are constantly exposed to microbes including their own microbiota, and a need for defenses against this threat including an immune system and all the epithelial barriers located at body sites bordering the environment seems obvious. At the same time, the advantage conferred by the extensive microbiota at these barriers is well-documented, and especially so for the largest of them, the intestinal microbiota (1). This includes the requirement during childhood of microbiota for normal development of the immune system. The intestinal barrier is not only a mechanical defense line but also serves as an advanced physiologic defense regulator balancing costs of the defense against its benefits. The barrier contains, in addition to epithelial cells, an intricate network of immune cells dominated by a special type of T cells and of neurons constituting the enteric nervous system (ENS). It is thus not surprising that the intestinal microbiota and barrier influence autoimmune disease (2–7). The aim of this opinion article is to make clear the vast scale of autoimmune disease complexity, and it argues that today’s focus on targeting specific activating components of adaptive immunity should be complemented with other approaches involving not the least the intestinal microbiota.

## The vast scale of autoimmune disease complexity

For a long time, the pathogenesis of autoimmune diseases was considered to be largely limited to malfunctioning adaptive immunity (8, 9). More lately, a more complex picture has emerged along with the recognition of the danger theory of immunity and recent insights into how the immune system interacts with, e.g., the central nervous system (CNS) and the microbiota (10–18). The presence within each of these complexity components of opposing stimulating and inhibitory activities eventually forming an optimum balance point contributes to the vast scale of this complexity of the disease pathogenesis. One example illustrating the wide repertoire of factors involved in the complexity is the anti-DNA autoantibodies typical for SLE penetrating into cell nuclei and inhibiting the DNA damage response (DDR) defense type (19, 20). Another example of the complexity of microbiota and adaptive immunity interactions is how bacteria escaping from the intestinal microbiota into the blood can elicit a cascade of defense events starting with production of inflammatory cytokines which, if left unchecked by regulatory activities, may cause septic shock. This defense involves the ‘inflammatory reflex’ initiated by bacterial endotoxin stimulating the vagal nerve and ending with inhibition of splenic macrophage cytokine secretion. The mechanism of the neurotransmitter acetylcholine (Ach) within this reflex was originally thought to be direct binding to macrophages, but activation by the splenic nerve of Ach-secreting CD4+ T cells is now considered to be more important (21, 22).

The critical role for many diseases (not only the autoimmune) played by the intestinal microbiota within the complex defense against dangers has been documented in detail for checkpoint immune therapy in cancer (23, 24). A ‘beneficial’ microbiota composition is a strong prognostic indicator that the therapy can cure cancer by pushing the physiological balance point towards more activity, releasing cytotoxic T cells from blockade exerted by Treg cells. This benefit is often accompanied by the cost of autoimmune adverse events; the pathologic balance point in most autoimmune diseases is characterized by too much activation and too little inhibition. There is preliminary evidence indicating a clinical potential against autoimmune diseases of pushing this balance in the opposite direction, including a nanomedicine-based novel tool (25–27). A critical role is supported also by some integrated omics data and a mechanistic mathematics-based approach documenting, for example, a bidirectional feedback interaction between intestinal microbiota and intraepithelial T cells (28, 29). This complex interplay involves modulation of microbiota composition by both adaptive immune cells and diet.

To summarize this outline on autoimmune disease complexity: it seeks to illustrate the overall message of this opinion article: complex regulatory activities within a network addressing commonly occurring dangers may cause autoimmune disease, and therapeutic modulation of this systemic network has a potential to be curative.

## Sjögren’s disease illustrates autoimmune disease complexity

Sjögren’s disease (SjD) can serve to illustrate the vast complexity of autoimmune disease because there is ample evidence that the patients display over-reactivity of a complex defense network including the immune system, the DDR, the nervous system and the gastrointestinal tract (8, 30–33). Immune system and DDR abnormalities overlap; the V(D)J recombinase complex participates in both repair of DNA damage and the DNA recombination needed for maturation of the adaptive immune system (30). Two of its components, Ku protein and DNA-dependent protein kinase catalytic subunit (DNA-PKcs), are targets of autoantibody formation and display increased activity in SjD cells (30).

Gastrointestinal abnormalities in SjD include dysbiosis of the microbiota and increased intestinal inflammation and permeability of the intestinal barrier (i.e., ‘gut leakage’) (2, 33). In order to further illustrate autoimmune disease complexity, in multiple sclerosis (MS), where the autoimmunity is considered to be limited to the CNS, there are no signs of intestinal inflammation, but MS shares with SjD both dysbiosis and a leaky gut. Thus, in MS the intestinal pathology may be secondary to CNS-mediated regulation of the ENS while in SjD it may be mediated by autoimmune destruction of intestinal barrier exocrine cells (33).

There is emerging evidence suggesting the possibility that anti-tumor defense can cause SjD and other forms of autoimmune disease (34). Some scleroderma patients have an antinuclear autoantibody to RNA polymerase III subunit C1 (RPC1) and in addition a cancer harboring a mutation in the RPCI *POLR3A* gene. This autoantibody reacts similarly with the mutant and the wild type form of the RPC1 protein, suggesting that the tumor triggered the patient’s autoimmune disease. It can be speculated that scleroderma patients with no clinical history of cancer may have benefitted from a stress response strong enough to eliminate a preclinical tumor (but at the same time causing this autoimmune disease). There is an odd observation indicating the relevance of this type of stress response in SjD; there is a markedly reduced incidence of some female cancer types including breast cancer in this the most women-dominated of all autoimmune diseases (35).

## Consequences of complexity for therapeutic modulation of intestinal microbiota and adaptive immunity

Treatment of autoimmune diseases spans a wide spectrum from molecule-specific targeting drugs to broadly acting glucocorticoids suppressing a number of physiologic processes (36). These regimens bring significant benefit by reducing morbidity and increasing life expectancy. Although our understanding of disease mechanism has greatly advanced, current therapy still emanates to a large extent from an old era, and most patients cannot be cured. This forms a parallel with another common disease, namely cancer, and autoimmune disease and cancer shares some important components of disease complexity: adaptive immunity and the intestinal microbiota (23, 37).

A basic feature of the complexity may be the regulation of the body’s attempt to restore homeostasis in the face of threats (38). Observations on autoimmune complexity indicate that it has much in common with how complexity is viewed within systems biology and chaos theory (39–41). Systems biology assumes that the whole of complexity is more than the sum of its components. A complicated system or problem like a human-made machine, e.g., a PET scanner, typically contains many components, with well-known properties and interactions. Stimulating or blocking a component has a predictable and reproducible effect. If disease complexity were such a problem, the current targeting strategy would be ideal. In contrast, complexity is ‘dynamic’ (meaning that it is continuously modified in many dimensions), and it is difficult to predict the effect resulting from manipulation of a specific component. Nevertheless, chaos theory suggests that many complex but seemingly stochastic biological phenomena follow some simple rules that allow for predictability of the consequences of such manipulation. If we find that our present view on autoimmune disease fits with this description of complexity, then it is not surprising that targeting a single or a few molecules will seldom lead to a cure.

To summarize this reflection on complexity and the current targeting approach to autoimmune disease: while the omics revolution most probably will continue to provide substantial clinical benefits, the vast scale of the complexity may turn out to be an unsurmountable challenge if we aim at a curative effect.

## Suggestions for future work on intestinal microbiota and adaptive immunity

The frontline of autoimmune disease therapy centers on inhibition of stimulatory targets within the immune system (36). However, it may be wise to consider that such targeting of specific network components may not be ideal for solving a complex problem. It might be worthwhile to explore the potential of activating the inhibitory arm of physiological stress responses or to look for molecules with an overarching regulatory role (12, 16, 28, 29). A predictable as well as precise effect, possibly conferring an extraordinarily sustained disease remission, by disabling the CD40 ligand (CD40L) (having nonredundant and far-reaching immunoregulatory effects) has recently been documented in SLE, SjD and MS patients (42). Releasing immunosuppressive Treg cells from brakes imposed by stress response regulation has been attempted (12) as well as a variant of checkpoint immunotherapy activating (instead of blocking) the inhibitory T cell surface PD-1 molecule (25–27). Teplizumab targeting the pan T cell CD3 surface molecule is documented to postpone the diagnosis of diabetes mellitus type 1 in high-risk children (43). The mechanism has been found to be regulatory, in the sense that it involves enhancing the suppressive T reg subset and exhaustion of activated autoimmune CD8+ T cells.

Another way to meet the challenge posed by complexity may be to combine several drugs targeting different stress response systems known to be involved in autoimmune disease.

There is a trend in the literature to explore the therapeutical potential of modulating the intestinal microbiota and the CNS, instead of only the immune system (5, 13–18). There is evidence pointing to the microbiota’s bidirectional feedback crosstalk with T cells, and to more complex contacts with nerve cells and metabolism (44, 45). One way to indirectly modulate the intestinal microbiota may be to reprogram the complex CNS – adaptive immunity – microbiota interplay (44) by means of targeting the Ach receptor on T cells (22). Along the same line of reasoning, a single targeting agent could be employed to modulate two major stress response types. One candidate such target is DNA-PKcs being part of both the immune system and the DDR. This candidate is supported by some recent findings. The first is on Graves’ disease and Hashímoto’s thyroiditis suggested to be caused by a stress response type named ‘autoimmune surveillance of hypersecreting mutants’ with T cells attacking somatically mutated thyroid cells and helping B cells to produce the pathogenic autoantibodies (46). Furthermore, it has been shown that an elevated level of DNA damage is a normal part of the exceptionally rapid proliferation rate displayed by T cells (47).

The architecture and regulating function of the intestinal barrier seems to be an important meeting point between the microbiota and host responses (48). Because of this complexity, it may be worthwhile to make an attempt to move beyond the currently dominating targeting strategy towards systemic modulation. There is preliminary data supporting that this is not merely a speculative idea. A novel method based on single cell integrated omics results has documented interactions between adaptive immunity and other cells in the intestinal barrier as well as throughout multiple organs (49, 50). The authors of one of these reports argue that intestinal epithelial lymphocytes therefore ‘represent intriguing but underexamined therapeutic targets for inflammatory diseases ….’ (49). Interestingly, a future research field may be to look for potential benefits conferred by well-known common drugs prescribed for other diseases than the autoimmune and influencing multiple organs and systemic factors such as hormones and metabolism (51).

The challenges with microbiota therapy can be illustrated by findings that each person has her own microbial ‘signature’, which is as specific as her fingerprint, and that the definition of a beneficial signature and of dysbiosis may vary between persons and types of autoimmune diseases (1, 9, 52, 53). Several ways to modulate the microbiota have been outlined, encompassing not only the transfer of feces but also diet and orally taken species thought to act beneficially (probiotics) (54). Safety and ethics aspects of treatments involving modulation of a patient’s intestinal microbiota’s personal ‘signature’ must be addressed. They were recently discussed in a report demonstrating unintended and persistent alterations of metabolism and immune regulation (15). In addition, a review on fecal microbiota transplantation concludes that the methodology needs to be improved due to rare but serious adverse events linked to infection caused by an intestinal microbiota species (55). An intriguing idea, while perhaps hard to realize, but counteracting many safety and ethics concerns, is for every healthy person to submit a fecal sample for a detailed metagenomic sequencing mapping. In the case a patient becomes diagnosed with an autoimmune disease, the intestinal microbiota could then be therapeutically modified in the direction of the patient’s known healthy signature. It is just possible that the beneficial intestinal microbiota is capable to persuade the complex stress response to return to the healthy balance point and thus achieve the cure.

## Conclusions

The vast scale of autoimmune disease complexity and its implications for development of future therapies are described. The great potential of modulating the intestinal microbiota’s crosstalk with adaptive immunity as well as other systemic factors such as the CNS and the DDR is outlined. It is hypothesized that alternatives to a targeting strategy, taking into consideration experiences within systems biology, might bring a curative effect.
